# Hypervascular Retained Products of Conception Mimicking Uterine Arteriovenous Malformation Following Surgical Termination

**DOI:** 10.51894/001c.166034

**Published:** 2026-08-01

**Authors:** Drew Warren, Steve Min

**Affiliations:** 1 College of Osteopathic Medicine Michigan State University

## 130

### Background

Delayed post-termination hemorrhage is an uncommon but potentially life-threatening complication that may be associated with retained products of conception or uterine arteriovenous malformation (AVM). Because these conditions require fundamentally different management strategies, accurate differentiation is critical to prevent inappropriate intervention and associated morbidity.

### Case Presentation

A 25-year-old G4P2113 presented with acute heavy vaginal bleeding approximately two months after surgical termination of pregnancy. She reported passage of large clots and bleeding severe enough to require pad changes twice per hour, with associated abdominal pain and dizziness. Initial hemoglobin on arrival was 12.2 g/dL and progressively declined to 6.4 g/dL throughout her hospitalization. Pelvic ultrasound demonstrated endometrial thickening with marked hyperemia and enlarged vessels at the endometrial-myometrial interface, raising concern for uterine AVM versus hypervascular retained products of conception. Due to the concern for AVM, angiography was performed; however, no evidence of vascular malformation was identified. The patient subsequently underwent hysteroscopy with dilation and curettage, during which abundant intrauterine tissue was removed. Histopathologic evaluation demonstrated retained chorionic villi consistent with retained products of conception. Postoperatively, the patient developed symptomatic anemia requiring intravenous iron therapy followed by transfusion of one unit of packed red blood cells. Her bleeding improved, and she was discharged in stable condition.

### Discussion

Hypervascular retained products of conception may closely mimic uterine AVM on Doppler imaging, creating diagnostic uncertainty. Misdiagnosis may result in delayed or inappropriate management and further complications. Accurate differentiation of these etiologies guides appropriate management and prevents unnecessary intervention.

### Conclusion

Delayed post-termination hemorrhage with hypervascular uterine findings requires careful diagnostic evaluation to distinguish retained products of conception from uterine AVM. Awareness of this diagnostic overlap is essential to avoid the potentially life-threatening complications associated with uterine arteriovenous malformation.

